# Coexistence of a primary ALK-positive and MET14 exon skipping mutation double-fusion in one patient with NSCLC and response to crizotinib: A case report and literature review

**DOI:** 10.1097/MD.0000000000047628

**Published:** 2026-02-20

**Authors:** Ke Xu, Minghui Wang, Jijing Zhao, Xiaofeng Xu, Meijun Song

**Affiliations:** a Department of Respiratory Medicine, The First People’s Hospital of Jiande, Hangzhou, Zhejiang, China; b Department of Clinical Medicine, Bengbu Medical College Graduate School, Bengbu, Anhui, China; c Department of Respiratory Medicine, The First People’s Hospital of Jiashan, Jiaxing, Zhejiang, China; d Department of Respiratory Medicine, Zhejiang Provincial People's Hospital Bijie Hospital, Bijie, Guizhou, China; e Department of Pulmonary and Critical Care Medicine, Zhejiang Provincial People’s Hospital (Affiliated People’s Hospital, Hangzhou Medical College), Hangzhou, Zhejiang, China.

**Keywords:** anaplastic lymphoma kinase, crizotinib, MET exon14 skipping mutation, non-small cell lung cancer, targeted therapy

## Abstract

**Rationale:**

Lung cancer exhibits one of the highest incidence and fatality rates globally. With the advancement of next-generation sequencing testing techniques, double or multiple gene driver mutations have been identified in certain patients.

**Patient concerns:**

A 78-year-old female presented with a chest shadow.

**Diagnoses:**

In this case of lung adenocarcinoma, second-generation sequencing revealed a co-occurrence of echinoderm microtubule-associated protein-like 4-anaplastic lymphoma kinase and MET exon 14 skipping mutation.

**Interventions:**

Crizotinib was administered orally on August 31, 2022, resulting in a partial response and progression-free survival for a duration of 8 months. After 8 months of treatment, the patient’s disease progressed, after which the treatment dose of crizotinib was increased; the patient’s condition improved again.

**Outcomes:**

Over 4 months of increased-dose oral crizotinib treatment, the patient achieved durable partial response, with significant reduction in tumor burden and without new metastases.

**Lessons:**

This report supports crizotinib can provide potential benefit for anaplastic lymphoma kinase/MET14 co-mutated lung adenocarcinoma patients, but sufficient cases and further research are needed to confirm and explore the possible mechanisms involved.

## 1. Introduction

Anaplastic lymphoma kinase (ALK) is a tyrosine kinase present in 3% to 7% of patients with non-small cell lung cancer (NSCLC) who have a fusion mutation in the ALK gene.^[1]^ The MET gene is one of the tumor driver genes for NSCLC, encoding the c-Met protein, which acts as a tyrosine kinase receptor for hepatocyte growth factor and plays crucial roles in embryogenesis, tumor growth, and metastasis.^[2]^ Studies have demonstrated that MET exon 14 skipping mutation accounts for approximately 3% to 6% of all NSCLC cases,^[3,4]^ and only about 3% to 4% of lung adenocarcinomas.^[3,5]^ Generally considered independent driver mutations in NSCLC, MET exon 14 skipping mutation are mutually exclusive with other driver mutations (epidermal growth factor receptor [EGFR], ALK, and ROS1), and are associated with poor prognosis.^[6]^ However, with the widespread use of next-generation sequencing technology, some patients have been found to harbor double or multiple gene driver mutations. The efficacy of ALK inhibitors in patients with co-mutations involving both ALK and MET14 remains uncertain.

Currently, few data exist on overlapping mutations in EGFR, KRAS, MET, and echinoderm microtubule-associated protein-like 4 (EML4)-ALK individuals. The interaction (synergism vs dominance) in the setting of 2 oncogenic driver alterations and response to tyrosine kinase inhibitors (TKI)-treatment is poorly understood. A cohort of 13 patients with co-occurring EGFR and ALK mutations revealed that mutant EGFR and ALK fusion proteins were co-expressed and co-localized within the same tumor cell clones, yet exhibited distinct phosphorylation patterns – suggesting differential activation states where one oncogenic alteration may potentially dominate over the other.^[7]^ Regarding ALK/EGFR co-alterations, Sabine Schmid et al demonstrated that EGFR-TKIs exhibits superior activity compared to ALK-TKIs. The intertumoral variability of ALK rearrangements and varying phosphorylation levels of oncogenic drivers may partially elucidate the differing response rates to ALK-TKIs compared to EGFR-TKIs in patients with ALK/EGFR co-alterations. Nonetheless, a separate study utilizing different molecular testing techniques indicated that a low EGFR mutation burden may explain the absence of clinical benefit from EGFR-TKIs in these individuals.^[8]^ The interplay, encompassing both synergistic and dominating effects, between 2 oncogenic driver changes, namely ALK and MET, and their influence on TKI therapy response remains inadequately comprehended. Due to the infrequency of these co-alterations in driver genes and the limited clinical experience with targeted TKIs, the present comprehension of their prognostic importance and predictive utility is considerably constrained. Charley Jang et al^[9]^ reported the first documented case of dual alectinib and capmatinib therapy in a patient with ALK rearranged NSCLC and METex14 mediated primary resistance. Combinatorial therapy continues to be a promising treatment modality for patients who exhibit acquired resistance mechanisms following TKI therapy. For patients who exhibit acquired resistance mechanisms after TKI therapy, combined therapy continues to be a promising treatment approach. Moreover, in contrast to monotherapy, initial treatment utilizing multi-kinase inhibitors and combination therapies that concurrently target ALK alongside MET, EGFR, and ROS1 may produce synergistic and more sustained responses while mitigating TKI resistance. Crizotinib, a multi-kinase inhibitor, may serve as a successful treatment for ALK-rearranged NSCLC exhibiting MET-mediated resistance. Recent studies indicate that patients who have acquired MET-driven resistance, such as MET amplification after next-generation ALK inhibitor therapy, may benefit from combined targeting of ALK and MET.^[10–12]^

Herein we report a rare case of advanced lung adenocarcinoma harboring an ALK/MET14 co-mutation and review relevant literature.

## 2. Case presentation

**Figure 1.**
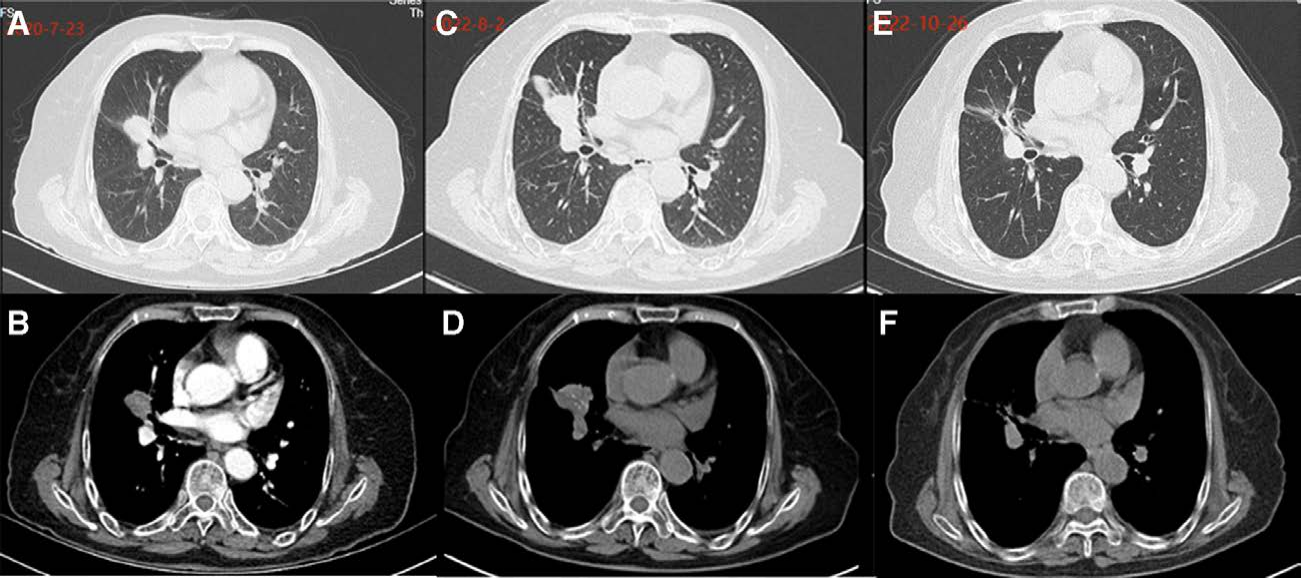
Comparison of CT images of both lungs before and after crizotinib treatment. (A and B) Neoplasm occupying the lateral segment of the right lung middle lobe. (C and D) Adenocarcinoma was diagnosed without treatment, and the disease progressed 2 years later. (E and F) After 2 months of treatment with crizotinib, the tumor was significantly smaller than before. CT = computed tomography.

**Figure 2.**
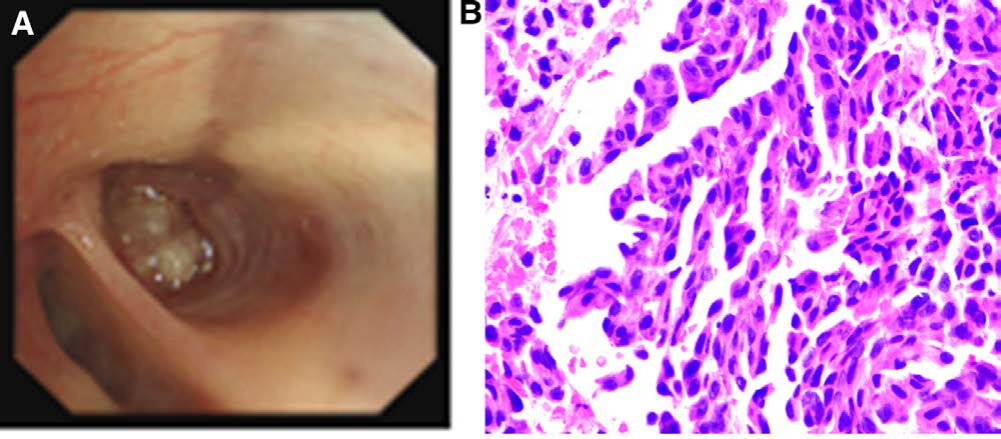
Bronchoscopy and pathological findings (HE staining, ×400). (A) Neoorganisms obstructed lumen in the right medial lateral segment. (B) The pathology of the new biopsies revealed adenocarcinoma.

**Figure 3.**
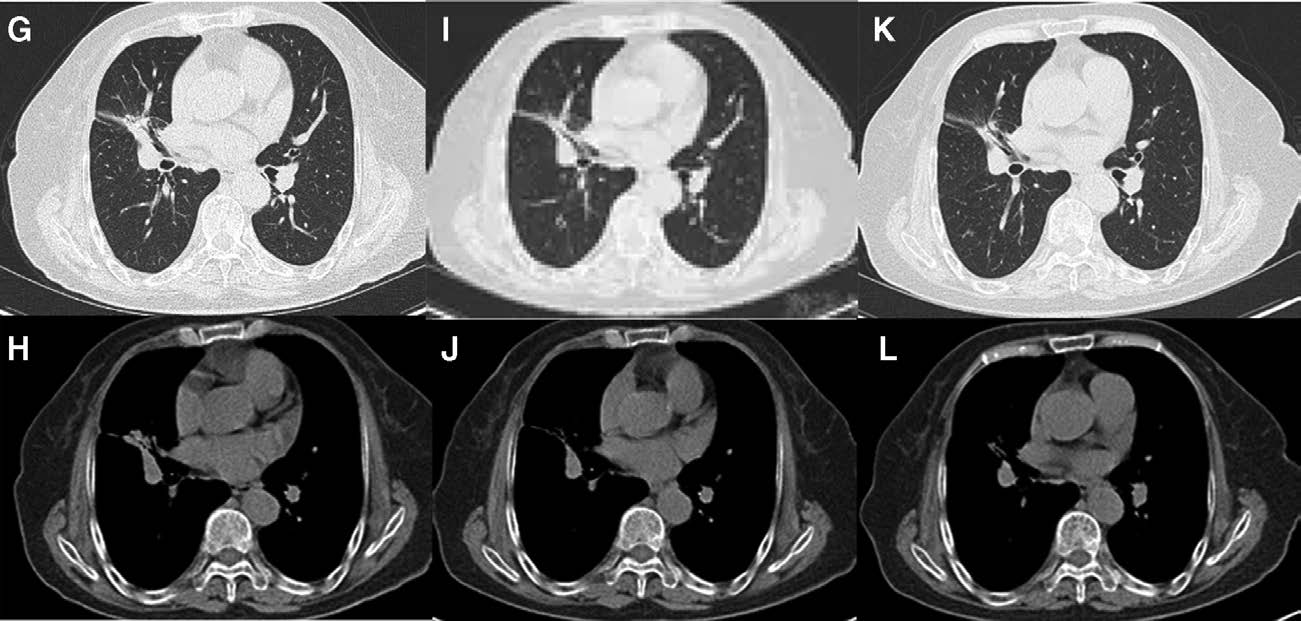
(G and H) Following 6 months with crizotinib, the patient is exhibiting progressive disease. (I–L) Restaging scans 2 and 4 months after increasing the therapeutic dose of Crizotinib treatment showed an excellent response radiologically.

The patient, a 78-year-old female, was admitted to Zhejiang Provincial People’s Hospital in July 2020 due to the discovery of pulmonary nodules for 2 years. The patient had no history of smoking and no family history of cancer. Chest computed tomography (CT) revealed obstructive pneumonia in the right outer middle lobe of the lung, which indicated a predisposition to lung cancer. A ground hyalinoid tubercle was observed at the posterior end of the upper lobe of the right lung (Fig. 1A, B). Bone scans and enhanced magnetic resonance imaging showed no significant abnormalities in the head region. Tracheoscopy identified a new organism obstructing the trachea in the lateral middle lobe of the right lung. Biopsy and brush examination were performed, with pathological analysis indicating adenocarcinoma (Fig. 2). Immunohistochemistry results showed positive expression for A1-1 NapsinA(+), TTF-1(+), CK7(+), while P40(−) did not exhibit fluorescence in situ hybridization. Further next-generation sequencing results from biopsy tissue suggested an EML4-ALK fusion and MET14 exon jump mutation. After complete staging examination, it was diagnosed as stage IIIA right lung adenocarcinoma with ALK-positive and MET exon 14 skipping mutation (T2N2M0). The patient was advised to continue treatment at hospital but chose self-discharge against medical advice. In August 2022, due to coughing and hemoptysis persisting for 2 days, she returned for further evaluation. Chest CT revealed enlargement of lung cancer in the lateral middle lobe of her right lung accompanied by new dense shadow formation inside (Fig. 1C, D). The patient received the first-generation ALK-TKI crizotinib on August 31, 2022, at a dose of 62.5 mg, bid standard daily dose, and responded well at first evaluation. On October 26, 2022, CT scan showed that lung cancer in the lateral middle lobe of the right lung was significantly smaller than previously estimated, and the efficacy evaluation showed partial response (PR; Fig. 1E, F). Outpatient follow-up to March 2023, CT review showed that after targeted therapy, the right lung cancer was fuller and denser than before, so the comprehensive evaluation was progressive disease (PD; Fig. 3G, H). At this point, the patient began oral crizotinib 125 mg bid targeted therapy in March 2023. Follow-up chest CT scans at 2 months and 4 months of increased oral crizotinib dosage showed that the patient’s tumor had shrunk compared to before, with a PR treatment response evaluation (Fig. 3I–L).

## 3. Discussion

**Figure 4.**
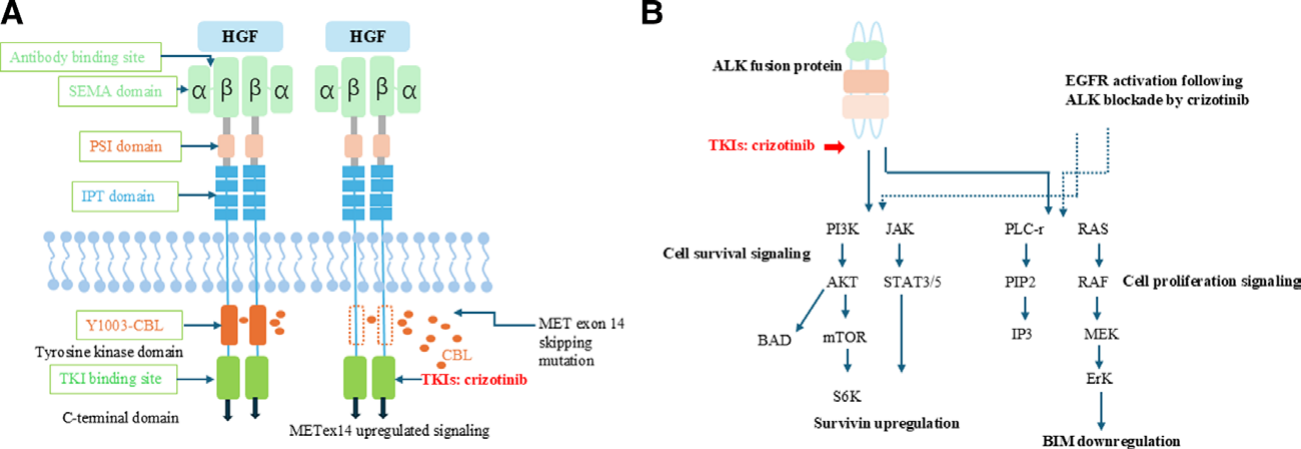
ALK and MET signaling pathway. (A) The mutations occurring in the METex14 flanking region led to abnormal splicing and exon 14 deletion, resulting in the generation of METRTK lacking ubiquitin binding sites. This leads to reduced ubiquitination and MET degradation, thereby causing an increase in stable expression and addiction to oncogenic genes. (B) ALK fusion mutations form a fusion protein with persistent activity, leading to the proliferation, survival and metastasis of tumor cells. ALK = anaplastic lymphoma kinase.

Currently, targeted therapy has revolutionized the treatment of NSCLC with driver gene mutations, ushering in the era of precision medicine and significantly prolonging patient survival while enhancing their quality of life. Crizotinib, a multi-target small-molecule tyrosine kinase inhibitor, has gained approval for its efficacy in ALK fusion-positive patients, ROS1 positive patients, as well as those with locally advanced or metastatic MET14 exon skipping mutations in NSCLC. The METex14 skipping mutation in NSCLC affects the coding segment of the near-membrane domain of RTK, thereby disrupting the normal regulation of the gene product. Exon 14 encodes a part of the near-membrane domain and contains Y1003 (a CblE3 ubiquitin ligase binding site). The ubiquitination of the MET receptor leads to its degradation, thereby controlling the expression of MET.^[13]^ Crizotinib competitively binds to the ATP-binding pocket of the MET receptor, occupying the ATP binding site and preventing ATP from binding to the MET kinase domain. This results in the inhibition of MET’s autophosphorylation and activation (Fig. 4A). The ALK gene frequently undergoes fusion mutations (such as EML4-ALK) in tumor cells, resulting in the formation of a fusion protein with persistent activity. Crizotinib, an ATP-competitive inhibitor, selectively binds to the ATP binding site of the ALK kinase domain, thereby obstructing the phosphorylation activation of the ALK fusion protein. This subsequently inhibits downstream signaling pathways (including MAPK, PI3K-AKT, and JAK-STAT), thereby suppressing tumor cell proliferation, survival, and metastasis (Fig. 4B).^[14]^ In ALK-positive patients, the PROFILE 1014 study demonstrated that first-line crizotinib treatment substantially extended median progression-free survival compared to chemotherapy (10.9 vs 7.0 months; HR = 0.454; 95% CI: 0.346 − 0.596; *P* < .0001), along with a significantly higher objective response rate (74% vs 45%; *P* < .0001).^[15]^ For patients harboring MET14 exon skipping mutations treated with crizotinib according to PROFILE 1001 trial data, the objective response rate was found to be 32.3%. The median duration of response was recorded at 9.1 months and the median progression-free survival stood at 7.3 months.^[16]^ Consequently, crizotinib may serve as an efficacious therapeutic approach for NSCLC possessing both EML4-ALK fusion and MET exon 14 skipping mutation. However, there is an absence of comprehensive clinical research regarding the efficacy of TKI in treating patients with concurrent ALK and C-MET gene double mutations. Some evidence indicates that patients with acquired MET-driven resistance such as MET amplification following next-generation ALK inhibitor therapy may derive clinical benefit from dual ALK/MET inhibition.^[10,11]^

This article summarizes the cases of ALK/MET co-mutated lung cancer that have been treated with targeted therapy, by searching the Chinese Biomedical Literature Database, Chinese Science and Technology Journal Database, Wanfang Database, PubMed, EMBASE, and other databases, GUI et al^[17]^ reported a case of locally advanced pulmonary mucinous adenocarcinoma with positive c-MET gene amplification combined with positive ALK fusion. The lung lesions were completely resolved after 1 month of crizotinib treatment. Zhu et al^[18]^ retrospectively analyzed the clinical data of 3 patients diagnosed with dual drive coexistence of c-MET and ALK. After treatment with crizotinib, the efficacy was evaluated as 1 case of PR, 1 case of stable disease, and 1 case of PD. These findings suggest that patients with ALK and C-MET dual driver mutations in lung adenocarcinoma may have poor response to TKI treatment. A retrospective study conducted by CHEN et al^[19]^ revealed that c-MET overexpression coexists with ALK rearrangement in a small subset of advanced NSCLC patients. This suggests that individuals with acquired MET mutations may potentially benefit from simultaneous targeted therapy for ALK/MET. Studies^[20]^ have indicated that the dual alteration response involving the ALK and C-MET genes diminishes the effectiveness of TKI treatment, although the difference is not statistically significant. Among the 3 patients with dual mutations who received crizotinib treatment, 2 had PD and 1 had stable disease. Here we report a rare case where double mutation involving ALK positivity and MET14 exon skipping was detected in primary lesions prior to molecularly targeted therapy administration, without any acquired resistance to TKIs. The majority of ALK-positive lung cancers acquire resistance independent of ALK alterations, often through activation of alternative or bypass signaling pathways,^[21–23]^ among which activation of the c-Met pathway is considered one mechanism underlying resistance to ALK-TKI therapy. A comprehensive analysis of MET alterations in ALK-positive NSCLC revealed that MET serves as an important bypass mechanism in tumors exposed to next-generation ALK TKIs,^[24]^ with MET amplification detected in one-third of patients treated with next-generation ALK TKIs, providing a rationale for exploring ALK/MET combination therapy as an initial treatment strategy for advanced ALK-positive NSCLC. However, the role of MET exon 14 skipping mutation in ALK-positive patients remains unclear. Basic studies indicated that the expression of ST7-MET in ALK-positive cell lines can induce resistance to ALK-TKIs, which can then be reversed by dual inhibitors of ALK/MET.^[25]^ Haoyue Hu et al^[25]^ reported a rare molecular subtype of NSCLC with simultaneous EGFR mutation and ALK rearrangement. The authors suggested that non-EML4-ALK co-alteration may be the mechanism of acquired drug resistance induced by EGFR-TKIs, while EML4-ALK co-alteration may be a neonatal change. In addition, there are 2 possible scenarios in which these 2 gene alterations coexist at the beginning of tumor proliferation. These 2 biomolecular alterations may co-exist in different cell clones, which represent heterogeneous expression of tumors or the same tumor cells. All mechanisms are possible and may occur at different times in the course of the disease in the same patient.^[26]^ Two different driver genes were found in this patient before treatment. Crizotinib, as a multi-target small molecule tyrosine kinase inhibitor, achieved good therapeutic effect in the treatment of this patient. Currently, there is no standard treatment plan for 2 or more co-mutations. Dong Qiu et al^[27]^ reported a case of advanced NSCLC harboring both an EGFR L858R mutation and a ROS1 fusion. The patient exhibited primary resistance to EGFR TKI therapy and was subsequently treated with a salvage regimen combining chemotherapy and Endostar. Current research indicates that the mechanisms of EGFR-TKI resistance are highly heterogeneous and primarily focus on acquired resistance, while co-activation of certain oncogenic signaling pathways may lead to uncontrolled proliferation or survival of lung cancer cells, thereby conferring intrinsic EGFR-TKI resistance. Ali Kaan Güren et al^[28]^ reported a case of HER2 exon 20 mutant NSCLC with complete remission of intracranial metastases with trastuzumab deruxtecan.

This interesting case verifies that ALK-positive and MET14 skipping mutations can achieve favorable outcomes under crizotinib treatment. Nevertheless, the interaction (synergism vs dominance) in the setting of 2 oncogenic driver alterations such as ALK and MET remains unclear. Prior to treatment the patient identified 2 separate driver genes, several distinct scenarios may exist: The mutant MET and ALK fusion proteins may be co-expressed and co-localized within the same tumor cell clone, displaying varying phosphorylation patterns and distinct activation states. In this scenario, one oncogenic driver may demonstrate a competitive advantage over the other through potential hierarchical signaling dominance. Alternatively, these molecular alterations might coexist in different cell clones, reflecting either intertumoral heterogeneity or intercellular variability within the same tumor mass. Furthermore, these genetic events could have emerged either synchronously or metachronously during disease progression. All of these mechanistic possibilities remain biologically plausible and warrant further investigation. Currently, with respect to 2 or more different molecular biological co-alterations, there is no standardized treatment protocol.

## 4. Conclusions

From the above cases and relevant literature both domestic and abroad, we found that crizotinib targeted therapy may be effective for NSCLC patients with ALK-positive and MET14 skipping mutations. When NSCLC develops acquired resistance mutations after first-line targeted therapy, it may still benefit from other targeted drug treatments. Further research is needed to confirm and explore the possible mechanisms involved.

## Author contributions

**Writing – original draft:** Ke Xu, Minghui Wang, Jijing Zhao, Xiaofeng Xu.

**Writing – review & editing:** Ke Xu, Meijun Song.
